# P-2015. Applying PEN-FAST in Clinical Practice: Using the PEN-FAST Decision Tool to Guide Penicillin Allergy Challenges in Hospitalized Patients

**DOI:** 10.1093/ofid/ofaf695.2179

**Published:** 2026-01-11

**Authors:** Caitlin Hart, Katherine C Shihadeh, Timothy C Jenkins, Margaret M Cooper

**Affiliations:** Denver Health, Evergreen, CO; Denver Health, Evergreen, CO; Denver Health, Evergreen, CO; Denver Health, Evergreen, CO

## Abstract

**Background:**

Penicillin allergies are frequently documented without reevaluation, often resulting in fewer antibiotic options and increased use of broad-spectrum agents. Recent evidence supports direct oral challenges for patients identified as low risk utilizing the PEN-FAST clinical decision tool. The PEN-FAST score was incorporated into a pharmacist-led penicillin allergy testing protocol to identify patients eligible for de-labeling. The purpose of this study was to assess the rate of penicillin allergy de-labeling and the incidence of adverse reactions following testing.

**Methods:**

This was a retrospective study of adults hospitalized at a 550-bed academic medical center with a reported penicillin allergy who had allergy testing performed between January 1st, 2019 and August 1st, 2024. Allergy testing included direct oral, 2-step oral, IV, 2-step IV, and skin testing. Variables collected include patient demographics, index penicillin allergy history, PEN-FAST score, type of allergy test performed, outcomes of the allergy test, rescue medications administered within 24 hours of allergy test, and change in allergy label.

**Results:**

Of 52 patients who had allergy testing, 40 (76.9%) had the allergy removed from their Electronic Health Record. A comment in the EHR stating the challenge was tolerated occurred in 10 (19.2%) patients. A penicillin allergy was re-added in 3 patients (5.8%) despite no new reactions post-challenge, and 1 patient (1.9%) retained their allergy label despite tolerating the challenge. The most common allergy test was the 2-step oral challenge (46.2%), followed by direct oral (34.6%), skin test (15.4%), and IV or 2-step IV challenge (1.9% each). Of the 13 patients with a PEN-FAST score of 3, none had a reaction. Of the 29 patients with a PEN-FAST score of 1 or 2, one (3.4%) had a reaction, reported as dyspnea and skin eruption after receiving a 10% test dose of amoxicillin and managed without significant harm. Of the 10 patients with a PEN-FAST score of 0, none had a reaction.

**Conclusion:**

Among hospitalized patients, a pharmacist-led protocol for low risk penicillin allergies resulted in the safe de-labeling of a substantial proportion of allergies. The PEN-FAST score proved to be a valuable tool in identifying low risk patients appropriate for allergy challenges.

**Disclosures:**

All Authors: No reported disclosures

